# Inhibition of Adipogenesis and Induction of Apoptosis and Lipolysis by Stem Bromelain in 3T3-L1 Adipocytes

**DOI:** 10.1371/journal.pone.0030831

**Published:** 2012-01-24

**Authors:** Sandeep Dave, Naval Jit Kaur, Ravikanth Nanduri, H. Kitdorlang Dkhar, Ashwani Kumar, Pawan Gupta

**Affiliations:** Institute of Microbial Technology (CSIR), Chandigarh, India; University of Tor Vergata, Italy

## Abstract

The phytotherapeutic protein stem bromelain (SBM) is used as an anti-obesity alternative medicine. We show at the cellular level that SBM irreversibly inhibits 3T3-L1 adipocyte differentiation by reducing adipogenic gene expression and induces apoptosis and lipolysis in mature adipocytes. At the molecular level, SBM suppressed adipogenesis by downregulating C/EBPα and PPARγ independent of C/EBPβ gene expression. Moreover, mRNA levels of adipocyte fatty acid-binding protein (ap2), fatty acid synthase (FAS), lipoprotein lipase (LPL), CD36, and acetyl-CoA carboxylase (ACC) were also downregulated by SBM. Additionally, SBM reduced adiponectin expression and secretion. SBM's ability to repress PPARγ expression seems to stem from its ability to inhibit Akt and augment the TNFα pathway. The Akt–TSC2–mTORC1 pathway has recently been described for PPARγ expression in adipocytes. In our experiments, TNFα upregulation compromised cell viability of mature adipocytes (via apoptosis) and induced lipolysis. Lipolytic response was evident by downregulation of anti-lipolytic genes perilipin, phosphodiestersae-3B (PDE3B), and GTP binding protein G_i_α_1_, as well as sustained expression of hormone sensitive lipase (HSL). These data indicate that SBM, together with all-trans retinoic-acid (atRA), may be a potent modulator of obesity by repressing the PPARγ-regulated adipogenesis pathway at all stages and by augmenting TNFα-induced lipolysis and apoptosis in mature adipocytes.

## Introduction

Adipose tissue is crucial for energy storage and lipid homeostasis [1], [2], [3], but an imbalance between energy intake and expenditure leads to obesity, which is a major risk factor for many chronic diseases and metabolic disorders such as type 2 diabetes, hypertension, hyperlipidemia, and arteriosclerosis [4]. Understanding the pathophysiology of obesity and finding treatment regimens are emerging areas of research. The preadipose 3T3 clonal cell line, 3T3-L1 [5], is a convenient cell culture model for both investigation of the program of adipogensis and determination of factors that regulate the physiology of the mature adipocyte. Quiescent cells can be induced to differentiate by the addition of various hormones or drugs in the presence of adipogenic factors that are in fetal bovine serum.

Adipose tissue mass is determined by processes governing adipocyte size and number [6]. The size of adipocytes increases because of increased storage of triacylglycerols from dietary sources or endogenous lipogenic pathways. Adipocyte number increases as a result of increased proliferation and differentiation [7]. Early in the adipogenic differentiation program preadipocytes undergo mitotic clonal expansion (MCE): a process that differs from proliferation of nonconfluent adipocytes and is essential for adipocyte differentiation [8]. MCE is accompanied by induction of CCAAT/enhancer-binding protein (C/EBP) β and C/EBPδ. During mid-phase adipocyte differentiation, the expression of CEBPα and PPARγ, which are both antimitotic, occurs as the cells exit the cell cycle. These proteins are thought to be terminating MCE [9], [10]. C/EBPα and PPARγ then coordinately drive expression of adipocyte-specific genes such as adipocyte fatty acid-binding protein 2 (aP2), fatty acid synthase (FAS), acetyl-CoA carboxylase (ACC), lipoprotein lipase (LPL), and cluster of differentiation (CD) 36, many of which characterize the final stages of differentiation [11]. The cAMP/glucocorticoid-triggered C/EBPβ pathway leading to PPARγ expression is now understood to be distinct from insulin stimulated Akt–tuberous sclerosis complex (TSC2)–mammalian target of rapamycin (mTORC) pathways leading to PPARγ expression [11], [12]. Decreases in adipose tissue mass may involve the loss of lipids through lipolysis and the loss of mature fat cells through apoptosis [13].

The clinical importance of herbal drugs and vitamins for treatment of obesity has received considerable attention [14], [15], [16]. These therapies have been variably efficacious on signaling pathways at early, middle, and late stages of adipocyte differentiation, and together with several other non-invasive dietary treatment regimens are desirable for this chronic disease. A number of herbal (flavonoid) and dietary inhibitors of adipose differentiation have been identified, including isorhamnetin [17], (-)-epigallocatechin-3-gallate (EGCG) [18], silibinin [19], retinoic acid [20], and 1,25(OH)_2_D_3_ [21].

Fruits such as papaya and pineapple (*Ananas comosus*) are also active constituents of an anti-obesity diet. Stem bromelain (SBM) [3.4.22.32], a widely accepted phytotherapeutic drug, is a member of the bromelain family of proteolytic enzymes obtained from *A. comosus* [22]. SBM is anti-edematous and anti-metastatic and also has several other therapeutic effects [23], [24], [25], [26]. In addition, it is being used efficaciously as an anti-obesity pill (France Vita Natura Pvt Ltd.). While its anti-metastatic effects and its modulation of immune cells have been adequately addressed [25], [27], [28], [29], its mechanism of action on adipocytes, however has not been addressed or understood. The diverse effects of SBM appear to depend on its unusual ability to traverse membranes and its acceptability as an orally administered enzyme with minimal side effects [24], [30], [31], [32], [33]. This has been confirmed by several Good Clinical Practice (GCP)-conforming clinical studies [26], [33], [34], [35], [36]. Because of its therapeutic use in treatment of obesity, it is crucial to understand the biological effects of SBM in the cell milieu.

The objective of this study was to elucidate the molecular mechanism of SBM modulation of adipogenesis using 3T3-L1 adipocytes as a model system. Interestingly, SBM modulates mid- and late-stage differentiation, but not MCE (early stage). It does so by selectively inhibiting the Akt phosphorylation and augmenting TNFα expression; these actions lead to repression of PPARγ and C/EBPα and their target genes aP2, LPL, FAS, ACC, and CD36, which are all involved in adipogenesis. The effects of SBM, unlike those of atRA and 1,25(OH)_2_D_3_, are irreversible and can occur post differentiation via the promotion of key events in apoptosis and lipolysis. These effects of SBM are dependent on its proteolytic activity. SBM and atRA treatment together was more potent in inhibiting adipocyte differentiation than any of the treatments alone.

## Materials and Methods

### Cell culture and differentiation

3T3­L1 mouse embryo fibroblasts was procured from cell repository at national centre for cell science, Pune, India and were cultured as described elsewhere (29). In brief, preadipocytes were cultured in Dulbecco's modified Eagle's medium (DMEM) containing 10% fetal calf serum until confluent. All media contained 1% penicillin–streptomycin (10,000 U/ml) and 1% (vol/vol) 100 mmol/l pyruvate. Cells were maintained at 37°C in a humidified, 5% CO_2_ atmosphere. For the differentiation of preadipocytes, two days after confluence, the cells were stimulated with DMEM containing 10% fetal bovine serum (FBS), 167 nmol/l insulin, 0.5 µmol/l isobutylmethylxanthine, and 1 µmol/l dexamethasone for 2 days. On day 2, the differentiation medium was replaced with 10% FBS/DMEM containing 167 nmol/l insulin. This medium was repleted every two days. Preadipocyte and adipocyte were treated with vehicle or test compounds in relevant media for time points as mentioned in figure legend. Total cell mass was assessed for different parameters.

### Oil Red O staining

Cells in 6-well/60 mm plates were washed twice with PBS and fixed for 10 min with 4% paraformaldehyde in PBS (pH 7.4). Cells were then stained for 30 min with Oil Red O (0.5 g in 100 ml isopropanol) as described by Suryawan and Hu [37]. In some wells, Oil Red O dye retained in the cell was quantified by elution into isopropanol, and OD_500_ was measured.

### GPDH activity assay

Cultured cells in 6-well/60 mm plates were treated with vehicle or test compound (10 µg/ml in PBS) along with differentiation media. Cells were washed twice with cold phosphate buffered saline (PBS) and collected by scraping with a cell scraper into 50 mM Tris-HCl (pH 7.5) containing 1 mM EDTA. The harvested cells were sonicated for 5 sec at 20% amplitude. After centrifugation at 13,000×*g* for 5 min at 4°C, the supernatants were assayed for GPDH activity. GPDH activity was determined spectrophotometrically by measuring the oxidation of NADH in the presence of dihydroxyacetone phosphate (DHAP).

### Quantification of triglycerides

Triglycerides were quantified by alkaline hydrolysis and measurement of released glycerol by the Free Glycerol Determination Kit (Sigma).

### MTT assay

Cells were cultured in a 96-well dish. After treatment for 24 h, a 20 µl aliquot of 3-(4,5-Dimethylthiazol-2-yl)-2,5-diphenyltetraolium bromide (MTT, a yellow tetrazole; 5 mg/ml in PBS) was added to the wells and incubated for 4 h at 37°C. The supernatant was removed carefully, 200 µl of DMSO was added and mixed, and the absorbance was read at 563 nm.

### Staining with annexin-V and PI

The annexinV-FITC Apoptosis Detection Kit (Calbiochem) was used to stain cells in annexin binding buffer according to the manufacturer's instructions. Cells were analyzed with a BD FACSCalibur flow cytometer (BD Biosciences). For quantification of % apoptotic cells, the cells were labeled with annexin V-FITC and six randomly selected fields were counted for fluorescent cells.

### TUNEL assay

Cell cultures were fixed with 4% paraformaldehyde, and in situ detection of cells with DNA strand breaks was performed by the terminal deoxynucleotidyl transferase dUTP nick end labeling (TUNEL) method using a Fluorescein FragEL™ DNA Fragmentation Detection Kit (Calbiochem) according to the manufacturer's instructions. Negative controls were performed by substituting Tris buffered saline (TBS) for the TdT enzyme. Cells labeled with the Fluorescein-FragEL DNA fragmentation detection kit (Cat No. QIA39) were analyzed with a BD FACSCalibur flow cytometer (BD Biosciences).

### Assay of DNA fragmentation

After treatment with vehicle or SBM, the cells were scraped and DNA was isolated as described elsewhere [38]. DNA fragments thus obtained were electrophoretically separated on a 1% agarose gel at 100 V for 30 min. The gel was stained with ethidium bromide and photographed under UV transillumination.

### ELISA of adiponectin

Adiponectin concentrations in cell supernatants were measured by enzyme-linked immunosorbent assay (ELISA). Adiponectin was captured in multiwall dishes by immobilized antibodies directed against mouse adiponectin. Captured protein was then detected by adding a second antibody against mouse adiponectin followed by a secondary anti-IgG antibody coupled to horseradish peroxidase (HRP). HRP substrate was added to the wells, and the intensity of the color change caused by the enzymatic reaction was measured at 450 nm.

### Cell proliferation assay

To assess cell proliferation, [^3^H]thymidine incorporation was measured after induction of differentiation of 3T3-L1 in the presence of 1 µCi of [^3^H]thymidine for 24 h. Briefly, cells were washed twice with PBS, once with ice-cold 10% TCA, and twice more with PBS. Cells were lysed in 500 µl of 2 N NaOH for over 6 h and samples were analyzed by a scintillation counter.

### Transactivation assay

The CD36 promoter, containing a PPARγ response element with the sequence 5′-AAGTCAGAGGCCA-3’, was cloned into the pGL3-Basic vector (Promega) by PCR-based cloning using a forward primer with an MluI restriction site and a reverse primer with a BglII site (creating a 300 bp promoter with a PPARγ response element) as mentioned elsewhere [39], [40]. The mutated PPARγ response element with the sequence 5′-AAGTCAGTTTCA-3′, for the CD36 promoter reporter was made by QuikChange Multi Site-Directed Mutagenesis Kit (Stratagene). The transactivation assay was performed using pGL3-CD36 promoter plasmid and an internal control (*Renilla* luciferase). Mature adipocytes (control and treated) were transiently transfected with a total of 500 ng pGL3-CD36 promoter plasmid and an internal control (*Renilla* luciferase). Relative luciferase activity was calculated by comparing the luciferase activity of the reporter construct with that of the internal control (*Renilla* luciferase). Transfection was done using Lipofectamine PLUS reagent (Invitrogen) according to the manufacturer's instructions. Normalized luciferase activities (relative light units; RLU) were plotted as the average (± SD) of data from triplicate wells from different cell culture.

### RT PCR

Cells were harvested in 1 ml of Trizol reagent (Invitrogen) and RNA was extracted according to the manufacturer's instructions. cDNA synthesis was performed with the RevertAid First Strand cDNA Synthesis kit (Fermentas) for RTPCR and RT-qPCR. RT-qPCR was performed in 96-well plates with the SYBR Green RT-qPCR kit (Invitrogen). PCR was performed in an iCycler iQ real-time PCR detection system, and the PCR baseline-subtracted data were computer generated as described by the manufacturer (Bio-Rad). 18S rRNA and β-actin were used as reference housekeeping genes for normalization. Primer details are available as Supporting information Table S1.

### Immunoblot analysis

Antibodies specific for β­actin, p-Akt, Akt, PPARγ, adiponectin, C/EBPα, C/EBPβ, and C/EBPδ, and all the secondary antibodies were from Santa Cruz Biotechnology (Santa Cruz, CA). Western blots were performed as described previously [28], [41].

### Reagents

SBM [EC 3.4.22.32] was obtained from Sigma. SBM solutions of native and catalytic-ally inactive were prepared, dialyzed extensively and subjected to size exclusion chromatography as mentioned elsewhere [28], [42], [43]. MTT assay reagents, Oil Red O, haematoxylin stain, insulin, β-nicotinamide adenine dinucleotide, dihydroxyacetone-3-phosphate, triethanolamine, atRA (purity ≥98% (HPLC)), 1,25-dihydroxyvitamin D3 (1,25(OH)_2_D_3_; vitamin D) (purity ≥99% (HPLC)), dexamethasone, and 3-isobutyl-1-methylxanthine were obtained from Calbiochem. DMEM, Trizol, fetal bovine serum, and penicillin–streptomycin were purchased from Invitrogen. Fetal calf serum was purchased from Hyclone. All chemicals not listed here were of analytical grade.

### Statistics

Results are expressed as the mean ± SD unless otherwise mentioned. SigmaPlot (SyStat Software) and SPSS (IBM) were used for statistical analysis. All statistical data were from averages of three or more independent experiments. Two-tailed Student t test was performed to obtain P values. Statistical significance was established at * P<0.01, ** P<0.05. The efficiency of PCR amplification for each gene was calculated by the standard curve method (E = 10^−(1/log slope)^). Gene expression was quantified by the comparative cycle threshold method (28), using 18S mRNA as an endogenous control. For relative mRNA abundance of C/EBPα C/EBPβ, C/EBPδ, and PPARγ, five different cell culture of SBM-inhibited adipocyte differentiation were considered in the study, and expression was calculated relative to control differentiated adipocytes after normalization to 18S rRNA.

## Results

### SBM inhibits 3T3-L1 adipocyte differentiation

**Figure 1:**
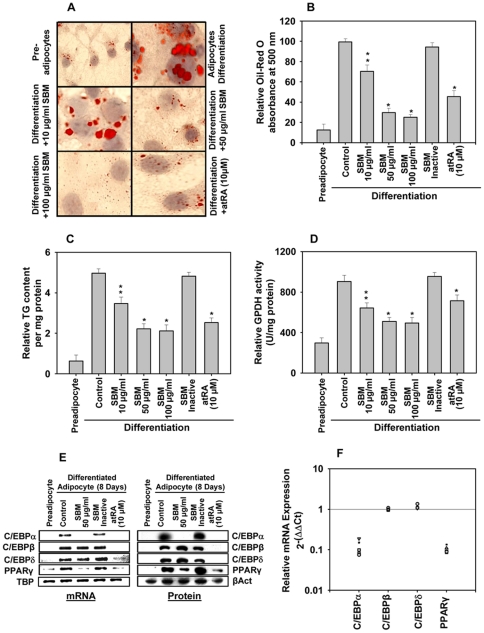
SBM inhibits adipocyte differentiation of 3T3-L1 cells. Two-day postconfluent 3T3-L1 preadipocytes (day 0) were treated with the indicated concentrations of SBM and was repleted every 2 days along with the relevant media cocktail upto day 8. Cells treated with 1X PBS were used as controls. The assays were performed on day 8. (A) Intracellular lipids were stained with Oil Red O. (B) Absorbance was spectrophotometrically determined at 500 nm after Oil Red O staining. (C) Triglyceride (TG) content (per mg protein) was measured with a triglyceride estimation kit (Sigma). (D) Glycerol-3-phosphate dehydrogenase (GPDH) activity (U/mg protein) was measured using a GPDH activity assay. (E) mRNA (RT PCR) and protein expression (western blot) of the adipogenic transcription factors C/EBPα, C/EBPβ, C/EBP-δ, and PPARγ. Results were expressed relative to untreated cells after normalization to TATA binding protein (TBP) and β Actin. (F) mRNA abundance of C/EBPα, C/EBPβ, C/EBPδ, and PPARγ in SBM-treated adipocytes relative to control differentiated adipocytes after normalization to 18S rRNA. Five biological replicates of SBM-treated adipocytes were tested. The results were confirmed by three independent experiments, which were each conducted in triplicate. Data are expressed as the mean ± SD. *P<0.01, **P<0.05 vs. controls.

To investigate the effect of SBM on adipocyte differentiation, we first looked at the accumulation of intracellular lipids. Confluent (day 0) 3T3-L1 pre-adipocytes treated with the inducing agents dexamethasone (Dex) and isobutyl methyl xanthine (IBMX) in the presence of fetal bovine serum and insulin, accumulate prominent lipid droplets as early as 4 days, however are more pronounced at day 8, and can be stained with Oil Red O. Treatment of cells with 50 µg/ml proteolytically active SBM during this time suppressed the number of colonies that stained for lipid (Figure 1A). This effect was 30% more pronounced than the inhibition caused by atRA, and it was not seen with the application of inactive SBM. While lower concentrations of active SBM were also effective in inhibiting differentiation, a saturating effect was seen at 50 µg/ml (Figure 1B). Short treatments with SBM (as short as 6 h) followed by a change of medium suppressed the adipocyte phenotype as effectively as when the drug was present continuously. This result, however, may reflect the difficulty of removing SBM from the cultures, due to its membrane-traversing ability [28], [32], and a minimal time requirement for SBM action cannot be assessed. For practical purposes, SBM was added to cultures during the treatment with Dex and IBMX, and it was maintained in the cultures when cells were switched to medium with fetal bovine serum and insulin alone.

To further characterize the extent of suppression of differentiation by SBM, estimates of cellular triglyceride (TG) content and glycerol-3-phosphate dehydrogenase (GPDH) activity were made, as these measures have been shown in most cases to correlate well with differentiation (Figure 1C and D). In both cases, values from SBM-treated dishes were ∼25% of control (untreated) values (TG, 22±3%; GPDH, 28±7%). The results from the GPDH assays were more variable and in some experiments appeared higher than expected, as compared to Oil Red O staining of replicate dishes. This could be because of the saturating activity of GPDH in cells that have been able to escape SBM-mediated inhibition of adipocyte cell differentiation.

We then investigated mRNA expression of the key genes C/EBPα, C/EBPβ, C/EBPδ, and PPARγ, which are involved in early- and mid-phase differentiation of adipocytes (Figure 1E, left panel). As expected, SBM reduced the mRNA level of C/EBPα and PPARγ, but interestingly, it did not affect the expression of C/EBPβ or C/EBPδ. Protein expression of these adipocyte markers, as measured by immunoblot, correlated with peak mRNA concentrations (Figure 1E, right panel). Relative expression (2^-(ΔΔCt)^) of mRNA as calculated from RT-qPCR corroborates the above (Figure 1F).

### SBM selectively inhibits mid- and late-phase adipogenesis

**Figure 2:**
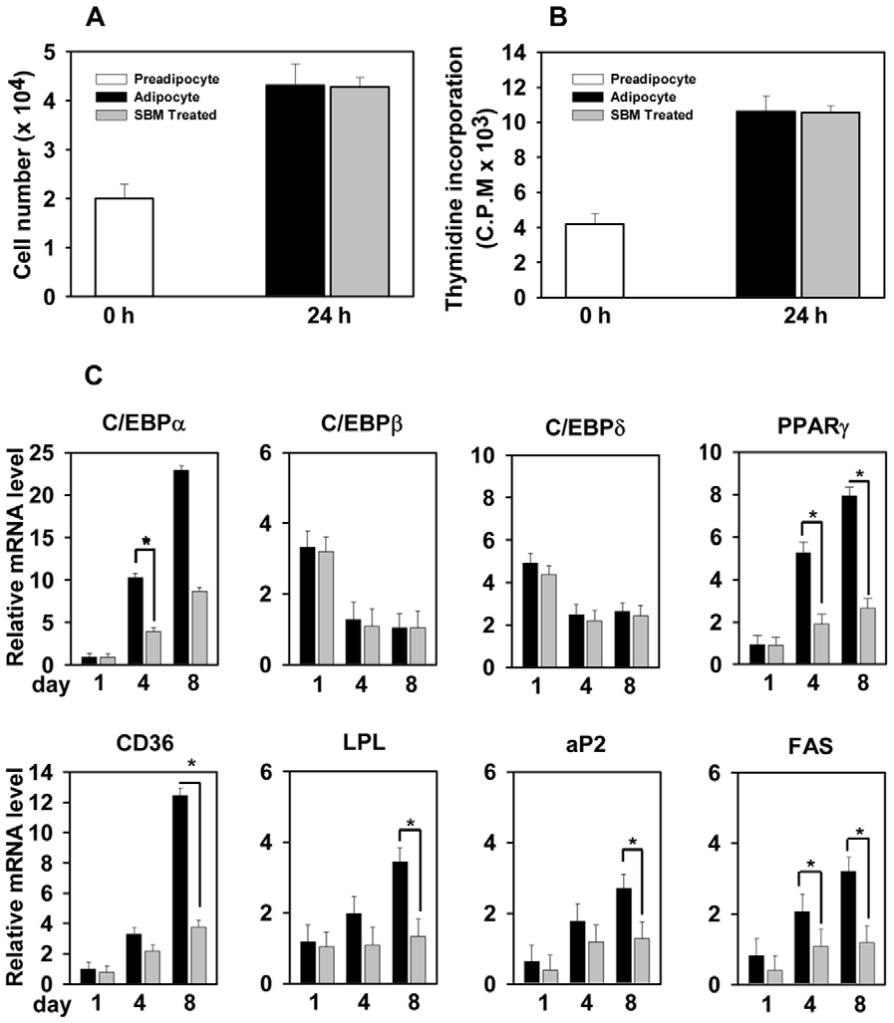
SBM does not inhibit mitotic clonal expansion. (A) Differentiation of 3T3-L1 cells was induced and cell number was counted in control and SBM-treated cells using a hemacytometer 24 h after induction. (B) Effect of SBM on DNA synthesis was monitored by [^3^H]thymidine incorporation after induction of differentiation of 3T3-L1 in the presence of [^3^H]thymidine for 24 h. Incorporation of [^3^H]thymidine into newly synthesized DNA was quantitated with a scintillation counter. (C) mRNA expression of adipogenic transcription factors C/EBPα, C/EBPβ, C/EBPδ, PPARγ, CD36, LPL, aP2 and FAS after 1, 4, and 8 days of differentiation in control and SBM-treated cells. Results were expressed relative to untreated cells after normalization to 18S rRNA. Total cell mass was assessed for different parameters. Data are expressed as the mean ± SD. *P<0.01, **P<0.05 vs. controls. The results were verified by four repetitions of the experiments, each of which was conducted in triplicate. Black bars  =  −SBM, gray bars  =  +SBM.

We next examined the effect of SBM (50 µg/ml) on early-, mid-,and late-phase differentiation. Early in adipogenesis, differentiation medium induces confluent pre-adipocytes to undergo one or two rounds of division (MCE), which is a prerequisite for differentiation into adipocytes. The number of preadipocytes increased almost twofold in 24 h after the addition of differentiation medium, in accordance with previous reports [44]. Interestingly, SBM had no effect on cell number or thymidine incorporation after induction of differentiation (Figure 2A and B). This result was expected, as SBM did not affect the expression of C/EBPβ or C/EBPδ, which are known to modulate MCE.

We then investigated mRNA levels of C/EBPα, C/EBPβ, C/EBPδ, and PPARγ, as well as aP2, FAS, LPL, and CD36. The latter proteins are markers of the final stages of adipocyte differentiation. They are target genes of C/EBPα and/or PPARγ. (Figure 2C). As expected, expression of C/EBPβ and C/EBPδ was limited to the early phase of differentiation and was not regulated by SBM. The expression of C/EBPα and PPARγ, however, was significantly reduced by SBM treatment, especially at late time points. Because SBM downregulated the expression of C/EBPα and PPARγ, we speculated that the expression of their target genes might also be downregulated. Indeed, under SBM treatment, the mRNA levels of aP2, FAS, LPL, and CD36 were notably reduced.

### Effect of SBM on 3T3-L1 cell viability and survival

**Figure 3:**
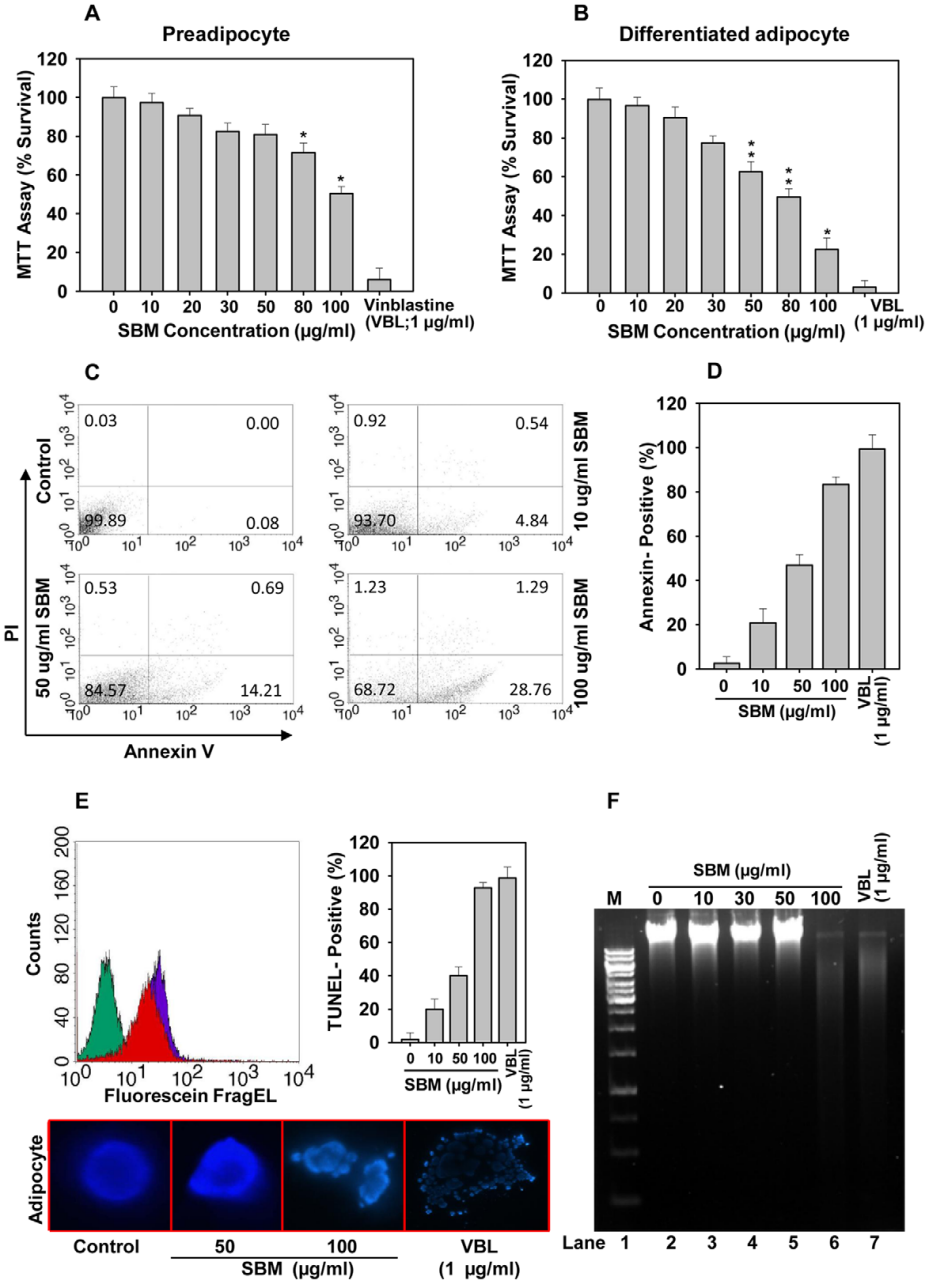
SBM induces apoptosis in mature adipocytes. Effect of SBM on the viability of, (A) 3T3L-1 preadipocyte and (B) mature adipocytes (8 days), as determined by MTT assay. Values are expressed as a percentage survival as compared to control after a 24 h incubation. (C) SBM-induced apoptosis in mature adipocytes evaluated by annexin-V/PI-FACS analysis. Annexin: FL1-H; PI: FL2-H. (D) Quantification of annexin V positive cells by imaging. At least 500 cells were analyzed. (E) Control (vehicle-treated) and SBM-treated mature adipocytes were either fixed and stained with DAPI and analyzed by fluorescence microscopy, or analyzed for DNA fragmentation by TUNEL. Cells labeled with the Fluorescein-FragEL were analysed by FACS shown, cell only (green) and cell treated with 100 µg/ml SBM (red), 1 µg/ml Vinblastine (purple) (left panel). Quantification of SBM-induced apoptosis in mature adipocytes by TUNEL assay (right panel). Micrographs of control and SBM-treated cells stained with DAPI are shown (bottom panel). (F) Demonstration of apoptosis by gel electrophoresis. Mature adipocytes were incubated with SBM at various concentrations for 24 h. Lane 1, DNA marker; lane 2, vehicle; lane 7, 1μM vinblastine positive control. Data are expressed as the mean ± SD. *P<0.01, **P<0.05 vs. controls. Results were verified by three repetitions of the experiments, which were each conducted in triplicate.

To determine whether SBM treatment affects cell viability and survival in 3T3-L1 preadipocytes and mature adipocytes, we performed an MTT assay, annexin–propidium iodide (PI) staining, a TUNEL, and a DNA fragmentation assay. Interestingly, while the MTT assay revealed only subtle changes in preadipocytes viability at all tested concentration of SBM, the viability of mature adipocytes at higher concentrations of SBM (100 µg/ml) was significantly reduced (Figure 3A and B). To determine whether the reduction in cell number was caused by apoptosis, annexin–PI staining was performed (Figure 3C). The annexin-PI staining combination assay detected apoptotic cell membrane phosphatidyl serine (PS) externalization and served as a measure of adipocyte viability. Whereas no significant increase in apoptosis was observed at 10 µg/ml and only a slight increase was observed at 50 µg/ml SBM concentration, a significant proportion of mature adipocytes underwent apoptosis at an SBM concentration of 100 µg/ml. Although annexin-PI staining is considered quantitative, it requires the segregation of cells for FACS analysis, and in that process may undercount some cells because of associated annexin V leaching. To address this issue, annexin V imaging was performed; it provided similar results but a more absolute quantitation (Figure 3D). To further investigate whether SBM-induced apopototic cells underwent DNA damage, we performed a TUNEL assay, which clearly showed increased labeling in SBM-treated cells (100 µg/ml SBM) compared to untreated cells (Figure 3E). Cells that stained positively in the TUNEL assay also showed DNA fragmentation with DAPI staining. The TUNEL assay and DAPI staining results are further supported by a DNA fragmentation experiment in which the genomic DNA of mature preadipocytes treated with SBM or vehicle was electrophoretically separated by agarose gel electrophoresis. DNA fragments obtained at 100 µg/ml SBM can be seen in the gel as a typical ladder migration (Figure 3F).

### SBM affects lipolysis by downregulating perilipin

**Figure 4:**
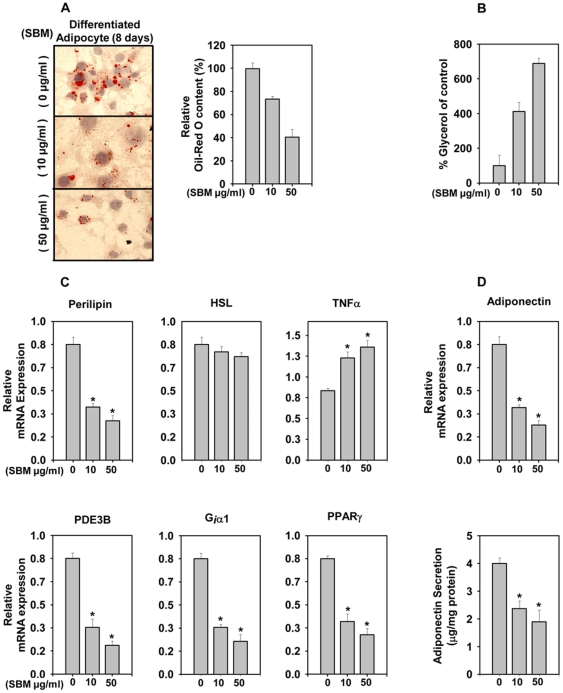
Increased expression of TNF-α-induces lipolysis in mature adipocytes upon SBM treatment. (A) SBM-treated (24 h, at 0, 10 and 50 µg/ml) mature adipocytes (8 days) were stained for intracellular lipids with Oil Red O. Retention of Oil Red O within cells was quantitated. (B) Glycerol release into the medium was measured in adipocyte treated with SBM (24 h, at 0, 10 and 50 µg/ml). (C) mRNA expression of the lipolysis-associated target genes perilipin, HSL, PDE3B, G_i_α_1_, TNFα, and PPARγ. (D) mRNA expression and secretion of adiponectin. mRNA expression was calculated relative to untreated mature adipocytes after normalization to 18S rRNA. Adiponectin protein in the media was measured by ELISA after treatment for 24 hrs at 0, 10 and 50 µg/ml SBM. Data are expressed as the mean ± SD. *P<0.01 vs. controls. The results were verified by three repetitions of the experiments, which were each conducted in triplicate. “− SBM”  =  cells treated with vehicle, “+ SBM”  =  cells treated with 50 µg/ml SBM.

SBM's effect on differentiated adipocytes (8 days) was monitored by Oil Red O staining (Figure 4A). A significant reduction in Oil Red O content was observed in 10–100 µg/ml SBM, suggesting a role for SBM in lipolysis. Free glycerol release was measured to get an estimate of lipolysis, which was significantly higher in SBM treated adipocytes (Figure 4B) at as low as 10 µg/ml and higher SBM concentrations. SBM's effect on lipolysis was assessed by measuring the expression levels of perilipin, phosphodiesterase-3B (PDE3B), GTP binding protein (G_i_α_1_)_,_ hormone-sensitive lipase (HSL), and tumor necrosis factor α (TNF-α) upon SBM treatment of mature adipocytes (post differentiation, 8 days) by real-time PCR. Treatment with SBM downregulated expression of perilipin, PDE3B, and G_i_α_1_, and upregulated the expression of TNF-α (and secretion; data not shown), but no significant changes were observed in expression of HSL (Figure 4C). SBM was also able to downregulate adiponectin expression and secretion (Figure 4D).

### SBM inhibits the expression of PPARγ by modulation of the Akt pathway

**Figure 5:**
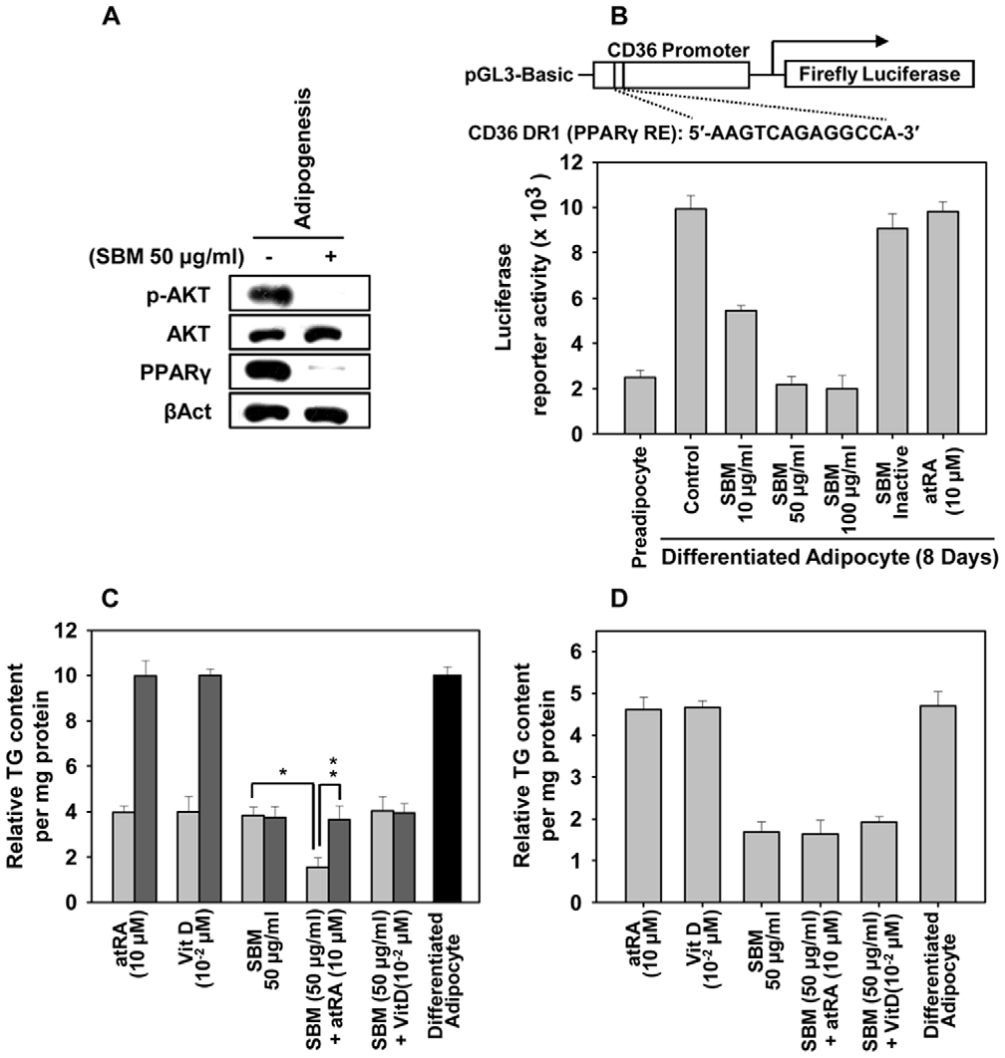
SBM is a potent and irreversible inhibitor of adipocyte differentiation. (A) SBM represses PPARγ by modulating the Akt pathway. Western blot of pAkt, Akt, and PPARγ in control and SBM-modulated differentiation. β-actin levels are shown as a loading control. Similar results were obtained for mature adipocytes (data not shown). (B) Estimate of PPARγ activity, as monitored by transfection of CD36 promoter luciferase reporter in mature adipocytes after 12 h of treatment with SBM. Cells were then harvested and lysed 12 h after transfection (24 h treatment with SBM). Luciferase activity is expressed as relative luciferase units after normalization. (C) Reversibility of inhibitors was monitored by first incubating the preadipocytes in differentiation medium containing 100 µg/ml SBM, 10 µM atRA, 10^−2^ µM 1,25(OH)_2_D_3_, or combinations of the three for 24 hrs (light grey bars); the medium was then replaced with fresh, inhibitor-free medium (dark grey bars), and lipid content was measured as TG content per mg protein. (D) SBM, unlike atRA and 1,25(OH)_2_D_3_, modulates mature adipocyte function. Mature adipocytes were treated with 100 µg/ml SBM, 10 µM atRA, 10^-2^ µM 1,25(OH)_2_D_3_, or combinations of the three for 24 hr, and lipid content was measured as TG content per mg protein. Data are expressed as mean ± SD. *P<0.01, **P<0.05 vs. controls. The results were verified by three repetitions of the experiments, which were each conducted in triplicate.

Because SBM does not modulate the transient cAMP/glucocorticoid-triggered CEBPβ pathways that contribute to early PPARγ expression [11], we suspected that SBM was involved in inhibition of PPARγ via an alternative mechanistic pathway triggered by Akt [12]. SBM treatment abrogates Akt phosphorylation in a pattern that correlates with PPARγ expression and without affecting Akt protein expression (Figure 5A). Akt phosphorylation regulates diverse biological processes [45], many of which could contribute to Akt's role in driving adipocyte differentiation. Indeed, the Akt–TSC2–mTORC1 pathway has been shown to regulate adipocyte differentiation by controlling PPARγ expression [12]. SBM upregulates TNFα in mature adipocytes, leading to repression of PPARγ expression. It seems that inhibiting the Akt pathway and increasing TNFα expression may contribute to PPARγ repression. To further explore SBM modulation of PPARγ expression, we measured activation of a CD36 promoter reporter containing a PPAR response element. While significant activation of the CD36 promoter reporter was observed for control mature adipocytes (as monitored by luciferase activity), CD36 promoter activity was completely lost in the SBM-treated cells (Figure 5B). These effects were specific to PPARγ activity as CD36 promoter with mutated PPARγRE failed to show responsiveness to all conditions tested (data not shown).

### SBM is an irreversible inhibitor of adipocyte differentiation that is more potent than atRA and 1,25(OH)_2_D_3_

We then investigated SBM's ability to inhibit adipocyte differentitation vis-à-vis vitamins that have been recommended for obesity disorder: atRA (the acid form of vitamin A) and 1,25(OH)_2_D_3_ (vitamin D). We also investigated the reversibility of SBM's effect on adipocyte differentiation. SBM, atRA, 1,25(OH)_2_D_3_, or combinations of the three were added to the differentiation medium of adipocytes at days 0–4 and then removed by changing to fresh SBM-free differentiation medium. As measured by TG content, SBM was a more potent inhibitor of adipocyte differentiation (Figure 5C and D). Furthermore, 3T3-L1 cells failed to differentiate into adipocytes after the change to fresh SBM-free differentiation medium, thus indicating that the inhibition of adipogenesis by SBM, unlike inhibition by atRA or 1,25(OH)_2_D_3_, is irreversible. SBM and atRA treatment together inhibited adipocyte differentiation more effectively than either alone (Figure 5C). Comparison of the effect of SBM, atRA, or 1,25(OH)_2_D_3_ treatments in differentiated adipocytes (at 8 days), indicated that only SBM was effective at reducing the TG content (Figure 5D). This result suggests that not only is SBM-inhibited differentiation irreversible, but SBM is also a more effective inhibitor of adipocyte differentiation after the differentiation is initiated. The time frame during which atRA or 1,25(OH)_2_D_3_ can inhibit adipocyte differentiation is limited to the period immediately following induction of differentiation with hormonal agents.

## Discussion

Because of SBM's therapeutic utility as anti-obesity alternative medicine, it is crucial to understand its biological effects in the cell milieu. In this study, we evaluated the effects of SBM on adipogenesis in mouse 3T3-L1 cells. Our results demonstrate that SBM irreversibly inhibits 3T3-L1 adipocyte differentiation by reducing adipogenic gene expression and that it induces apoptosis and lipolysis in mature adipocytes.

At the molecular level, adipogenesis is regulated by a complex transcriptional cascade that involves the sequential activation of C/EBPs and PPARγ [46]. C/EBPβ and C/EBPδ are rapidly and transiently expressed after the hormonal induction of differentiation cocktail, and C/EBPβ is required for MCE in the immediate early stages of adipocyte differentiation [47]. These temporally expressed transcription factors are induced and activated by cAMP and glucocorticoids and act synergistically to induce the expression of C/EBPα and PPARγ, the master adipogenic transcription regulators [11], [48]. C/EBPα and PPARγ, in turn, promote terminal differentiation by activating the transcription of the battery of genes involved in creating and maintaining the adipocyte phenotype. Our results indicate that exposing 3T3-L1 preadipocytes to SBM during adipogenesis reduces the level of C/EBPα and PPARγ mRNA, but that it does not affect the expression of C/EBPβ and C/EBPδ (Figure 1E and F). Therefore, SBM suppression of the upregulation of C/EBPα and PPARγ occurs independently of C/EBPβ gene expression. Recent studies of 1,25(OH)_2_D_3_, isorhamnetin (a flavonoid from seabuckthorn), and 18-alpha-glycyrrhetinic acid (AGA) showed that they reduced the level of C/EBPα and PPARγ mRNA, but did not affect the expression of C/EBPβ [17], [21], [49]. Conversely, gelsolin (an actin regulatory protein) and phloretin (a flavonoid in apples) have been shown to promote adipocyte differentiation by upregulation of C/EBPα and PPARγ without affecting the expression of upstream regulators C/EBPβ and C/EBPδ [50], [51]. These results are clearly suggestive of alternative and independent pathways leading to activation and repression of PPARγ. Recently, the Akt–TSC2–mTORC1 pathway has been shown to stimulate PPARγ expression and adipogenesis [12]. In our study, SBM was able to repress expression of PPARγ target genes ap2, FAS, LPL, CD36, and ACC (Figure 2C). Additionally, SBM reduced adiponectin expression and secretion, an effect that may contribute to decreased adipocyte differentiation. Interestingly, SBM seems to reduce Akt phosphorylation in adipocytes. SBM has been shown previously to reduce Akt phosphorylation in many other cell lines such as fibroblast, epidermoid carcinoma, melanoma, and skin papilloma [28], [29], [52]. Overall, the findings of this study shed light on a potential mechanism behind the inhibitory effect of SBM on adipocyte differentiation.

In many cells, including adipocytes, the Akt signaling cascade leading to NFkB activation is an important signal for cell survival [53]. Because SBM treatment reduces Akt phosphorylation without reducing Akt protein, we investigated adipocyte viability by MTT assay. SBM significantly reduced the viability of mature adipocytes but not preadipocytes (Figure 3A and B), which is understandable, as terminally differentiated lineages have more defined signaling and have distinct signatures [11]. Interestingly, SBM induced TNFα expression in mature adipocytes (Figure 4). SBM induction of TNFα has been reported previously in mononuclear cells [54], [55]. The reduction in adipocyte viability was most apparent at an SBM concentration of 100 µg/ml and is due to apoptosis but not necrosis, as apparent by annexin–PI staining, TUNEL assay, and DNA fragmentation assay (Figure 3C–F). Adipocyte apoptosis is an important mechanism for regulating adipocyte cell number, and its regulation is important in obesity-combating strategies [56]. SBM-induced apoptosis could be implicated in SBM's ability to block Akt signaling (Figure 5A) and induce TNFα expression (Figure 4C). TNFα-induced apoptosis involves binding to TNF receptor 1, which results in recruitment of TNF receptor-associated death domain (TRADD) and activation of the cascade of caspases that leads to cell death and phagocytosis by macrophages [57]. The TNFα pathway in adipocytes is also known for selective deletion of adipocytes but not preadipocytes [58], which can be ascribed to SBM's ability to selectively induce adipocyte but not preadipocyte apoptosis. These effects were similar even for longer time frames tested (data not shown). TNFα can also suppress expression and function of PPARγ [59], and this effect, combined with SBM's ability to reduce Akt phosphorylation, may contribute to repression of PPARγ by SBM.

The cytokine TNF-α is also an important mediator of lipid metabolism and also plays a role in inducing lipolysis [60]. TNF-α can perturb the normal regulation of energy metabolism, and enhanced TNF-α expression could be a cause as well as a consequence of the decrease of lipidic depots in white adipose tissue, the inhibition of insulin action, and the promotion of apoptosis [61]. Therefore, we evaluated the lipolytic response to SBM by measuring the expression levels of perilipin, HSL, and TNF-α during adipocyte differentiation (Figure 4C). SBM downregulated perilipin while upregulating TNF-α, but no appreciable changes were observed for HSL expression. PPARγ is known to upregulate perilipin expression [62], whereas TNF-α is known to promote phosphorylation and downregulation of perilipin [63]. The TNF-α induction and PPARγ repression caused by SBM can explain why SBM treatment causes perlipin downregulation. PPARγ impact on glucose uptake, metabolism and lipogenesis is well documented and as such its repression by SBM may contribute to reduced lipogenesis while enhancing lipolysis. TNF-α-induced lipolysis is also known to downregulate anti-lipolytic genes PDE3B and G_i_α_1_, and our results were consistent with the published reports [64], [65]. TNFα leads to both physiological apoptosis and lipolysis as is clearly evident from target gene expression. Further while apoptosis is marked at 100 µg/ml SBM concentration, lipolysis is clearly evident at concentration as low as 10 µg/ml, where no significant apoptosis is observed suggesting that these are independent physiological processes. However at higher concentration 50–100 µg/ml we cannot rule out lipolysis due to cell death and such contributions cannot be practically dissected. So far it is amply clear that SBM effector functions in adipocytes leading to inhibition of adipogenesis and induction of apoptosis at higher concentration and lipolysis even at concentration as low as 10 µg/ml are governed by its ability to inhibit Akt while augmenting TNFα pathway. Also, that these effects are dependent on its proteolytic activity. While it is amply clear that SBM catalytic activity is required for inducing these effects, intriguingly catalytically active papain (member cyteine protease) failed to show significant effect. This suggest that either this could be due to specificity in SBM catalysis or may have structural contributions as well. SBM proteolytic actions on surface receptors of immune cells have been addressed [25], [27]. It remains to be seen how SBM's proteolytic activity is required for its role in inhibiting adipocyte differentiation and if the above pathways could be mechanistically linked.

SBM's ability to modulate adipogenesis compared to natural vitamins atRA and 1,25(OH)_2_D_3_ was evaluated, because these vitamins have proven, mechanistically addressed roles in adipogenesis. SBM, unlike atRA and 1,25(OH)_2_D_3_, was able to inhibit adipogenesis irreversibly, and it also modulated adipocyte function post-differentiation (Figure 5C and D). atRA and 1,25(OH)_2_D_3_ reversibly inhibit adipocyte differentiation and, in the case of atRA, only early in adipogenesis. They do not modulate the function of mature adipocytes [21], [66]. This could explain failure of atRA to modulate CD36 promoter reporter activity and may be implicated to absence of signaling effectors that mediate atRA regulation of PPARγ expression [66], [67]. Treatment of adipocytes with SBM and atRA together was more potent than treatment with either of them alone or in other combinations. This result could be due to complementation by atRA of SBM's effects at the level of MCE, which SBM does not modulate.

**Figure 6:**
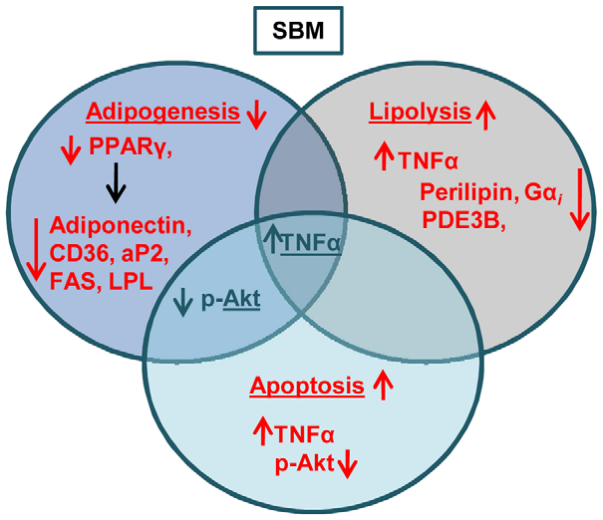
Schematic of SBM modulation of adipogenesis and adipocyte function. SBM blocks adipocyte differentiation and promotes lipolysis and apoptosis in mature adipocytes by increasing TNF-α expression and reducing Akt phosphorylation. While TNFα leads to enhanced lipolysis, increased apoptosis, and repressed PPARγ expression, reducing Akt phosphorylation leads to only the latter two.

Many attempts have been made to correct the metabolic disparity that is involved in obesity, using reagents such as sibutramine (an appetite suppressor), orlistat (a gastrointestinal lipid inhibitor), and fibrates (PPAR-α agonists) [68], [69]. Administration of these drugs, however, frequently causes undesirable side effects such as a dry mouth, anorexia, constipation, insomnia, dizziness, and nausea [70]. These negative effects have led to a high demand for therapeutically potent, yet safe anti-obesity drugs. SBM's acceptability as an orally administered enzyme and bioavailability with minimal side effects [24], [30], [31], [32], [33] have been confirmed by several Good Clinical Practice (GCP)-conforming clinical studies [26], [33], [34], [35], [36]. Our *in vitro* experimental data indicate that SBM may be a potent modulator of obesity by repressing the PPARγ-regulated adipogenesis pathway and augmenting the TNFα-induced lipolytic and apoptotic pathway (Figure 6). This naturally occurring phytotherapeutic may be beneficial for reducing diet-related obesity via its ability to regulate adipocyte differentiation. Further evaluation by *in vivo* experiments need to be done to support the therapeutic use of SBM as an anti-obesity nutritional herbal supplement. It will be interesting to examine whether the use of herbal formulations of SBM other than anti-obesity pill (France Vita Natura Pvt Ltd.) with quercitin, vinegar, hydroxyl citric acid (HCA), green tea, and guggulipid extract can be extended for obesity disorder.

## Supporting Information

Table S1 List of primer used in the study.
(DOC)
