# Supplementary material for: Inhibition of Adipogenesis and Induction of Apoptosis and Lipolysis by Stem Bromelain in 3T3-L1 Adipocytes
Source: PLoS One. 2012 Jan 24;7(1):e30831. doi: 10.1371/journal.pone.0030831 (PMC3265525; doi:10.1371/journal.pone.0030831)
Supplement: Table S1 — List of primer used in the study. (DOC) [file pone.0030831.s001.doc]

| **Gene Name** | **Forward primer** | **Reverse primer** |
| --- | --- | --- |
| Adiponectin | TCCTGGAGAGAAGGGAGAGAAAG | TCAGCTCCTGTCATTCCAACAT |
| aP2 | CATGGCCAAGCCCAACAT | CGCCCAGTTTGAAGGAAATC |
| CD36 | TCGGATCTGAAATCGACCTT | CACAGGCTTTCCTTCTTTGC |
| C/EBPα | AGCAACGAGTACCGGGTACG | TGTTTGGCTTTATCTCGGCTC |
| C/EBPβ | GGGGTTGTTGATGTTTTTGG | CGAAACGGAAAAGGTTCTCA |
| C/EBPδ | TTCCAACCCCTTCCCTGAT | CTGGAGGGTTTGTGTTTTCTGT |
| FAS | CTGAGATCCCAGCACTTCTTGA | GCCTCCGAAGCCAAATGAG |
| G*iα*_1_ | GCTTGCGGGAGCTGAGGACG | GCGAGCATCATCCGCCCGAG |
| HSL | ACAGTGCAGGTGGGAATCTC | GCCTAGTGCCTTCTGGTCT |
| LPL | GGCCAGATTCATCAACTGGAT | GCTCCAAGGCTGTACCCTAAG |
| PDE3B | ATGAGGAAAGACGAGCGCGA | CGGCAGAGGTGGAAGAAGAA |
| Perilipin | AGAGTTCTGCAGCTGCCTGTG | CAGAGGTGCTTGCAATGGGCA |
| PPARγ | CGCTGATGCACTGCCTATGA | AGAGGTCCACAGAGCTGATTCC |
| TBP | ACCCTTCACCAATGACTCCTATG | ATGATGACTGCAGCAAATCGC |
| TNFα | CCTGTAGCCCACGTCGTAG | GGGAGTAGACAAGGTACAACCC |
| 18S rRNA | CTTAGAGGGACAAGTGGCG | ACGCTGAGCCAGTCAGTGTA |

**Supplementary Table S1**

**Table S1-** List of primer used in the study. Abbreviations are explained below.

aP2, adipocyte fatty acid binding protein; C/EBP α, β and δ, CCAAT/enhancer-binding protein; CD36, Cluster of differentiation 36 molecule; LPL, lipoprotein lipase; FAS, fatty acid synthase; G_i_α_1_, GTP binding protein; HSL, hormone-sensitive lipase; PDE3B, phosphodiestersae-3B; PPARγ, peroxisome proliferative-activated receptor, gamma; perilipin, lipid droplet associated protein; HSL, hormone-sensitive lipase; TBP, TATA binding protein; TNFα, tumor necrosis factor alpha; 18S rRNA, 18S ribosomal RNA. 18S rRNA and TBP were used as the internal control.
